# Associations of motor and neuropsychiatric symptoms with comorbidities in prodromal Parkinson’s disease

**DOI:** 10.3389/fnagi.2024.1452766

**Published:** 2024-11-25

**Authors:** Jia-Ru Chen, Yan Sun, Yu-Ju Ma, Lan Tan

**Affiliations:** Department of Neurology, Qingdao Municipal Hospital, Qingdao University, Qingdao, China

**Keywords:** comorbidity, multimorbidity, Parkinson’s disease, motor symptoms, neuropsychiatry symptoms, multimorbidity patterns

## Abstract

**Objective:**

To investigate the associations between comorbidities and multimorbidity patterns with motor and neuropsychiatric symptoms in patients with Parkinson’s disease (PD) in prodromal PD.

**Methods:**

Multimorbidity is defined as the coexistence of two or more long-term conditions (LTCs) (also known as multiple comorbidities). A total of 921 participants without PD were included in the Parkinson’s Progression Markers Initiative (PPMI) database and were categorized according to the LTC count. Participants were evaluated on motor and psychiatric symptoms. Pearson correlation to examine relationship of comorbidities and target symptoms. The baseline population was analyzed using Multiple linear regression model, while mixed effects model was utilized for longitudinal analysis. Fuzzy C-means clustering analysis was conducted to identify comorbidity patterns, followed by multiple linear regression for further analysis.

**Results:**

At baseline, a higher LTC count was significantly correlated with more severe motor (MDS-UPDRS I, II, ADL, all *P* < 0.05) and neuropsychiatric symptoms (QUIP, *P* < 0.001). Three multimorbidity patterns were identified. Among them, the cardiometabolic multimorbidity pattern (CAR) had the most significant correlation with the aforementioned symptoms. Our longitudinal analysis indicated that an increase in the LTC count was associated with the exacerbation of motor and neuropsychiatric symptoms.

**Conclusion:**

Comorbidities were cross-sectionally and longitudinally associated with the motor and neuropsychiatric symptoms of patients with prodromal PD. Among the three multimorbidity patterns, CAR posed the highest threat to the risk of more severe motor and neuropsychiatric symptoms.

## 1 Introduction

Parkinson’s disease (PD) is the most common motor disorder and the second most common neurological disease, with its incidence and prevalence steadily increasing with age (GBD 2015 Disease and Injury Incidence and Prevalence Collaborators, 2016; Cui et al., 2021). PD is initially diagnosed according to the motor symptoms of the disease (Sveinbjornsdottir, 2016). Recently, PD has come to be understood as a systemic disease presenting not only motor symptoms but also non-motor symptoms, such as cognitive and neuropsychiatric symptoms. PD can be defined as a complex neuropsychiatric disease. More and more research evidence has shown that the incidence rate and severity of its neuropsychiatric symptoms often increase with the passage of time. These neuropsychiatric symptoms include anxiety, depression, impulse control disorders, autonomic nervous symptoms, etc. They can present in isolation but are frequently comorbid.

Up to 90% of senior people aged 60 or above suffer from two or more chronic diseases (Grande et al., 2021). Multimorbidity is defined as the coexistence of two or more long-term conditions (LTCs) (also known as multiple comorbidities). Comorbidities are common in patients with PD, and there is a high likelihood of interactions between different drugs for comorbidities, such as anticholinergics and benzodiazepines (Leelakanok and D’Cunha, 2019). In the late stage of PD, as the LTC count significantly increases and the use of concomitant medications becomes more frequent over time, comorbidities predict higher mortality rates (Santos Garcia et al., 2017). Furthermore, it is reported that several acquired comorbidities increase the risk of developing PD, such as hypertension, dyslipidemia, and diabetes mellitus (Potashkin et al., 2020). They may affect dopamine cells to disrupt the pathway between substantia nigra neurons and the putamen (Qiu et al., 2011), thus leading to an elevated risk of PD. They also can increase the risk of PD via chronic inflammation and oxidative stress. Comorbidities can increase the burden of brain pathology, leading to cerebrovascular damage or promoting neurodegeneration through interactions with neurons or synapses at the cellular level, further increasing the risk of PD (Vassilaki et al., 2016). Some chronic diseases usually do not cluster randomly. Concurrent chronic diseases might share common underlying risk factors (Fan et al., 2022). Another possibility is that one chronic disease arises out of another disease or the treatment of another disease. Identifying multimorbidity patterns will prevent and treat PD from a new point of view, which facilitates a better prognosis.

Previous studies mainly included patients with clinically diagnosed PD (Santos Garcia et al., 2017). There is a lack of studies on the associations of motor and neuropsychiatric symptoms with comorbidities in prodromal PD participants. Our study fills the gap. This study aimed to (1) investigate whether the LTC count is related to motor and neuropsychiatric symptoms of PD; (2) whether increased LTC count increases the likelihood of developing PD in the prodromal PD participants; (3) identify multimorbidity patterns in prodromal PD patients; (4) To assess the associations between different multimorbidity patterns and motor and neuropsychiatric symptoms of PD; (5) give suggestions for PD prevention from the perspective of comorbidities and multimorbidity patterns.

## 2 Methods

### 2.1 Participants

This study utilized data from the Parkinson’s Progression Markers Initiative (PPMI) database. Prodromal PD is defined as people who are at risk for Parkinson’s disease based on clinical characteristics, genetic variants, or other biomarkers but have not yet been formally diagnosed. The PPMI is an ongoing prospective, observational, international multicentered study. Our institutions were not involved in the PPMI data collection, which was downloaded from https://www.ppmi-info.org/data on 5th December 2022. The PPMI investigation was approved by the ethics review committees of all partner institutions; In addition, all subjects signed a written informed consent before enrollment.

A total of 2,121 patients who provided disease information were included in the PPMI database. After excluding PD patients (*N* = 866) and participants with missing data on age, gender, or educational years (*N* = 334), we included 921 participants who had already undergone evaluation of motor and neuropsychiatric symptoms of PD on various scales in the PPMI, consisting of prodromal PD patients (614 individuals), healthy controls (229 individuals), scan without evidence of dopaminergic deficit (SWEDD) (62 individuals), and early imaging (original study participants only, 16 individuals). None of the participants had been diagnosed with PD. The scales used in the PPMI were comprised of the Movement Disorders Society Unified Parkinson’s Disease Rating Scale (MDS-UPDRS) I, MDS-UPDRS II, MDS-UPDRS III, MDS-UPDRS IV, and the Schwab and English Activities of Daily Living (ADL) for motor symptoms, as well as the Geriatric Depression Scale (GDS), the Questionnaire for Impulsive Common Disorders in Parkinson’s Disease (QUIP), the Scale for Outputs for Parkinson’s Disease autonomous function (SCOPA-AUT), and the State Train Anxiety Inventory (STAI) for neuropsychiatric symptoms. The total numbers of our participants who underwent the evaluation without missing data for each scale were as follows: MDS-UPDRS I (*N* = 916); MDS-UPDRS II (*N* = 916); MDS-UPDRS IV (*N* = 148); ADL (*N* = 727); STAI (*N* = 863); GDS (*N* = 863); QUIP (*N* = 918); SCOPA-AUT (*N* = 857). Since only a small number of subjects underwent MDS-UPDRS IV (*N* = 148) (Supplementary Table 1), we did not consider this scale, with the remaining seven scales included in our study.

### 2.2 Clinical assessments

This study included data from 921 non-PD participants, with general information including gender, age, and education level. In addition, complete disease information was provided during the baseline and the whole 5-year follow-up period, and the arrange follow-up was conducted every year. The information provided included demographics and the type of disease in the population. All subjects were tested for motor and neuropsychiatric at the baseline and during the 5-year. MDS-UPDRS I assesses non-motor experiences of daily living, including cognitive impairment, hallucinations, depression, anxiety, apathy, etc. MDS-UPDRS II evaluates motor experiences of daily living (Goetz et al., 2007; Goetz et al., 2008), MDS-UPDRS IV is “motion complications” (Regnault et al., 2019). The ADL scale is used to assess the patients’ activities of daily living ability and the severity of disability and movement disorder (Cui et al., 2021). GDS, QUIP, SCOPA-AUT, and STAI were used to evaluate depression, impulse control disorders (ICDs), autonomous symptoms (AS), and anxiety in prodromal PD patients, respectively (Cui et al., 2021). MDS-UPDRS I was also assessed for cognitive impairment, hallucinations, depression, anxiety, apathy, and dopamine dysregulation (Goetz et al., 2007). The higher the ADL score, the stronger the independence and the smaller the dependency, which is negatively correlated with the number of comorbidities. Except for ADL, all the other scales were positively correlated with PD symptoms.

### 2.3 Comorbidities

We extracted comorbidities from medical records at the time of diagnosis. Comorbidities are recorded in the free text, and the existence of the following 21 chronic diseases was evaluated: hypertension, hyperlipidemia, diabetes, cardio HD, atrial fibrillation, cardiac valve diseases, obesity, sleep disorder, depression or anxiety, osteoporosis, degenerative joint disease, inflammatory arthropathy, thyroid diseases, solid neoplasms, anemia, eye disease, stomach disorder, chronic pancreas, chronic liver disease, COPD, autoimmune. The selection of these chronic diseases represents risk factors that may interfere with PD, and the aim is to explore the impact of the number of concurrent chronic diseases on motor and neuropsychiatric symptoms in participants with prodromal PD. The subjects were divided into three groups (488 patients in groups 0 to 1, 275 in groups 2 to 3, and 158 in groups ≥ 4). Patients with two or more chronic diseases are defined as multimorbidity (*N* = 433) (Fan et al., 2022). In the analysis of multimorbidity patterns, the population should first suffer from comorbidity, so the population with the number of comorbidity 0∼1 should be excluded, and the rest of the population should be de-extremalized.

### 2.4 Statistical analysis

We used Shapiro–Wilk to verify the normality test of the data are all non-normal distribution. Continuous variables were represented as means and standard deviations (SDs), and intergroup differences were evaluated using the Kruskal Wallis rank sum test. Using percentage representation for categorical variables and chi-square analysis to evaluate inter-group differences. Firstly, we studied the relationship between the number of comorbidities and physical and neuropsychiatric symptoms. Participants were divided into 0–1, 2–3, and ≥ 4 groups based on the number of comorbidities. Multiple linear regression models were used to study the number of comorbidities (independent variables) and the symptom scale scores (continuous, dependent variables) mentioned above. The covariates for all relevant analyses include gender, age, and education level. Next, the mixed effects model was used to describe the longitudinal relationship between comorbidities and behavior of daily living, anxiety, depression, ICDs, and AS in non-PD populations. We conducted a 5-year follow-up on the motor and neuropsychiatric scale of the population. Specifically, we used the number of comorbidities as the independent variable and the scores on the exercise and neuropsychiatric health scale as the dependent variable to analyze the correlation between the total population and each group. The covariates included age, gender, and education and compared the results with and without covariates. A mutually exclusive comorbidity category has been created for participants with chronic diseases of 0 or 1, 2 or 3, 4 or 4 or more. There is no or only one disease group as the reference group.

For the study of comorbidity patterns, fuzzy C-means clustering analysis in soft clustering analysis was used to generate clustering, which allows for the clustering individuals based on their underlying combinations of chronic diseases. The fuzzy c-means algorithm assigns a probability of cluster membership for all individuals within each cluster; however, participants were finally allocated a single cluster based on their highest membership probability (Aerqin et al., 2024). To explain the randomness of the clustering solution, 100 independent clustering repeats were conducted, and the average final result was generated. In the multimorbidity patterns, excluding diseases with a prevalence rate of less than 2% in the population can avoid noise and false findings in the pattern. Finally, 11 diseases were selected from the included chronic diseases. Participants are then assigned to clusters where they have the highest probability of membership, making it possible for patients in different patterns to share common diseases. By observing the expected ratio and disease exclusivity, the disease patterns of each group are examined. The former refers to the prevalence of a specific disease in a certain group divided by its prevalence in the total population, while the latter is defined as the proportion of participants in the disease included in the cluster to the total number of participants in the disease. When the observed/expected ratio is ≥ 2 or the exclusivity is ≥ 25%, the disease is considered to be associated with a given cluster, and name these patterns based on the characteristics of these standards (Guisado-Clavero et al., 2018; Aerqin et al., 2024).

A two-tailed *p*-value < 0.05 was considered statistically significant in all analyses. All statistical analyses were conducted using R 4.2.2 software (R Project for Statistical Computing)^1^ and IBM SPSS Statistics 25. Use the “lm” and “lmerTest” packages from R version 4.2.2 software for data analysis, as well as “ggplot2,” “ggsci,” “pheatmap,” “forestplot” in R version 4.2.2 software, Adobe Photoshop 2020 and Adobe Illustrator 2022 software for image production.

## 3 Results

### 3.1 Study participants

**FIGURE 1:**
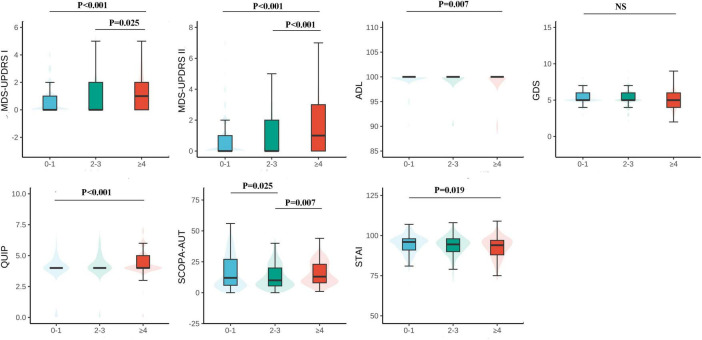
Motor and psychiatric scale stratified by the number of chronic diseases and their intergroup comparison. The level of MDS-UPDRS I, II, ADL, GDS, QUIP, and SCOPA-AUT increases along with the number of chronic diseases increases, however, ADL decreases. MDS-UPDRS, Movement Disorders Society Unified Parkinson’s Disease Rating Scale; ADL, Activities of Daily Living; GDS, Geriatric Depression Scale; STAI, State-Trait Anxiety Inventory; QUIP, Questionnaire for Impulsive-Compulsive Disorders in Parkinson’s Disease; SCOPA-AUT, the Scale for Outcomes for Parkinson’s Disease—autonomic function.

This study included 921 non-Parkinson patients, and the specific number of participants in each motor and psychiatric symptom scale is shown in the flowchart (Supplementary Figure 1). Data can be obtained from the basic information of subjects, motor and neuropsychiatric symptom scale scores, and the number of comorbidity information (Table 1). The mean age of the subjects was 63.15 ± 8.98 years old, and the average years of schooling was 16.47 ± 3.56 years. The proportion of females was 483 (52.4%), and there was no statistically significant difference in education attainment of the 3 groups (*p* = 0.081). Patients in the comorbidity groups (Patients with two or more chronic diseases) were older than those in the non-comorbidity group (suffering from 0 to 1 diseases) (*P* < 0.001), and the scores of motor symptoms (MDS-UPDRS I *F* = 6.488 *P* < 0.001; MDS-UPDRS II *F* = 18.120, *P* < 0.001; ADL *F* = 6.019 *P* = 0.007) and neuropsychiatric symptoms (QUIP *F* = 6.624 *P* < 0.001) were significantly different (Figure 1). In the multimorbidity patterns study, 488 subjects were excluded due to non-multi-disease status and 17 participants were excluded because of de-externalization. A total of 416 people met the final criteria. The basic information of patients in comorbidity mode can be seen in Supplementary Table 2.

**TABLE 1:** Baseline characteristics of participants by number of chronic conditions.

Variables (Mean ± SD)	Total (*N* = 921)	Number of chronic conditions			*P*	*β*
		**0–1 (*N* = 488)**	**2–3 (*N* = 275)**	**≥ 4 (*N* = 158)**		
Age	63.15 ± 8.98	62.07 ± 9.59^a^	63.32 ± 8.36^a^	66.17 ± 7.18^b^^c^	< 0.001	
Sex, *n* (%)					0.01	
Male	438 (47.6)	217 (44.5)	129 (46.9)	92 (58.2)		
Female	483 (52.4)	271 (55.5)	146 (53.1)	66 (41.8)		
Education, years	16.47 ± 3.56	16.27 ± 3.59	16.50 ± 3.67	17.01 ± 3.20	0.081	
MDS-UPDRS I (*N* = 916)	1.03 ± 1.66	0.85 ± 1.48^a^	1.13 ± 1.81^a^	1.43 ± 1.81^b^^c^	< 0.001	0.108
MDS-UPDRS II (*N* = 916)	1.63 ± 3.15	1.27 ± 2.54^a^	1.61 ± 3.25^a^	2.80 ± 4.24^b^^c^	< 0.001	0.292
ADL (*N* = 727)	98.63 ± 4.38	99.15 ± 3.08^a^	98.27 ± 5.47	97.52 ± 5.63^b^	0.007	−0.342
GDS (*N* = 863)	5.28 ± 1.31	5.33 ± 1.24	5.26 ± 1.33	5.15 ± 1.47	0.097	−0.028
QUIP (*N* = 918)	4.01 ± 1.50	3.84 ± 1.55^a^	4.09 ± 1.41	4.38 ± 1.40^b^	< 0.001	0.112
SCOPA-AUT (*N* = 857)	16.01 ± 12.62	17.26 ± 13.93^c^	13.75 ± 11.27^a^^b^	16.16 ± 9.87^c^	0.004	−1.24
STAI (*N* = 863)	93.90 ± 6.74	94.37 ± 6.58^a^	93.74 ± 6.85	92.64 ± 6.93^b^	0.016	−0.406

Data are summarized as mean ± standard deviation (SD) for continuous data and count (%) for categorical data. Data were missing for MDS-UPDRS I (*N* = 5, 0.5%), MDS-UPDRS II (*N* = 5, 0.5%), ADL (*N* = 194, 21.1%), GDS (*N* = 58, 6.3%), QUIP (*N* = 3, 0.3%), SCOPA-AUT (*N* = 64, 6.9%), STAI (*N* = 58, 6.3%);

*^a^*Significantly different from ≥ 4 group;

*^b^*Significantly different from 0 to 1 group;

*^c^*Significantly different from 2 to 3 group. *N*, number; MDS-UPDRS, Movement Disorders Society Unified Parkinson Disease Rating Scale; ADL, Activities of Daily Living; GDS, Geriatric Depression Scale; STAI, State-Trait Anxiety Inventory; QUIP, Questionnaire for Impulsive-Compulsive Disorders in Parkinson’s Disease; SCOPA-AUT, the Scale for Outcomes for Parkinson’s Disease—autonomic function.

### 3.2 Baseline correlation between multimorbidity and motor and psychiatric symptoms

The analysis revealed a significant correlation among the number of comorbidities and PD exercise and neuropsychiatric symptoms. Specifically, as the number of comorbidities increases, patients with MDS-UPDRS I (*β* = 0.108, *P* < 0.001), MDS-UPDRS II (*β* = 0.292, *P* < 0.001), QUIP (*β* = 0.112, *P* < 0.001) score also increased accordingly; The ADL (*β* = −0.342, *P* = 0.007) score decreased with increasing number of comorbidities. It can be considered that the number of comorbidities in prodromal Parkinson’s patients has an impact on motor and psychiatric symptoms of patients, and compared with 0∼1 comorbidity, patients in the group of ≥ 4 comorbidities had a greater impact on exercise and psychiatric symptoms of Parkinson’s disease (Table 1 and Figure 1). At baseline, the more patients had comorbidities, the more severe the ICDs in neuropsychiatric symptoms. However, contrary to expectations, STAI (*β* = −0.406, *P* = 0.016) and SCOPA-AUT (*β* = −1.24, *P* = 0.004) scores decreased in patients with a higher number of comorbidities. At baseline, GDS (*β* = −0.028, *P* = 0.097) did not correlate with the number of comorbidities. However, due to MDS-UPDRS I (*β* = 0.108, *P* < 0.001), there was a specific correlation between comorbidities and neuropsychiatric symptoms such as depression and anxiety. To verify the above results, we conducted a sensitivity analysis on the prodromal PD population in the non-PD population (Table 2). The results showed that in MDS-UPDRS I (*β* = 0.012, *P* = 0.044), MDS-UPDRS II (*β* = 0.021, *P* = 0.002), and QUIP (*β* = 0.016, *P* < 0.001), the scores also increased with the increase of comorbidities.

**TABLE 2:** Associations of motor and neuropsychiatric symptoms with comorbidities in prodromal Parkinson’s disease.

	*P*	*β*	OR (95% CI)
MDS-UPDRS I (*N* = 610)	0.044	0.012	1.012 (1.000, 1.024)
MDS-UPDRS II (*N* = 610)	0.002	0.021	1.022 (1.008, 1.035)
ADL (*N* = 611)	0.303	−2.87E-04	1.000 (0.999, 1.000)
STAI (*N* = 548)	0.046	−0.002	0.998 (0.997, 1.000)
GDS (*N* = 548)	0.040	−0.004	0.996 (0.991, 1.000)
QUIP (*N* = 611)	< 0.001	0.016	1.016 (1.008, 1.023)
SCOPA-AUT (*N* = 591)	0.028	−0.016	0.984 (0.969, 0.998)

### 3.3 Clinical trajectories of patients with different comorbidities

The longitudinal relationship between comorbidity status and prodromal PD motor and psychiatric symptoms was shown in Table 3. The more comorbidities the participants had, the faster the MDS-UPDRS II (*β* = 0.054, *P* < 0.001) score, SCOPA-AUT (*β* = 0.267, *P* < 0.001) score, GDS (*β* = 0.014, *P* = 0.035) score increase, and the faster the ADL (*β* = −0.237, *P* < 0.001) score decrease. After correction, the results are robust (Table 3). The above results support the impact and prediction of comorbidities on the motor and psychiatric symptoms of prodromal PD. In MDS-UPDRS I, QUIP, and STAI, this research didn’t find a longitudinal relationship between comorbidities and the symptoms reflected in the above scales. Longitudinal studies can determine that comorbidities were a dangerous factor for motor and psychiatric symptoms in participants with prodromal PD. This also confirms the baseline viewpoint. However, this does not mean that comorbidities have no longitudinal correlation with the above symptoms, and further exploration is needed.

**TABLE 3:** Longitudinal relationship between comorbidities and motor and neuropsychiatric symptom scale scores.

	Covariate correction			No covariate correction		
	***β***	***P***	**OR (95% CI)**	***β***	***P***	**OR (95% CI)**
MDS- UPDRS I	0.011	0.200	1.011 (0.994, 1.02)	0.011	0.183	1.012 (0.995, 1.028)
MDS- UPDRS II	0.054	0.003	1.055 (1.020, 1.090)	0.052	0.003	1.053 (1.019, 1.088)
ADL	−0.237	< 0.001	0.789 (0.669, 0.909)	−0.226	< 0.001	0.798 (0.679, 0.917)
GDS	0.014	0.035	1.014 (1.001, 1.027)	0.014	0.036	1.014 (1.001, 1.027)
QUIP	0.009	0.295	1.009 (0.992, 1.026)	0.007	0.410	1.007 (0.990, 1.024)
SCOPA-AUT	0.267	< 0.001	1.305 (1.191, 1.420)	0.292	< 0.001	1.339 (1.223, 1.454)
STAI	−0.006	0.861	0.994 (0.929, 1.059)	0.015	0.653	1.015 (0.951, 1.079)

Association of comorbidities with motor and neuropsychiatric symptoms in patients with pro-Parkinson’s disease at 5-year follow-up; Covariate correction: Use age, sex, and education level as covariate correction; No Covariate correction: age, sex, education correction is not used.

### 3.4 Multimorbidity patterns

**FIGURE 2:**
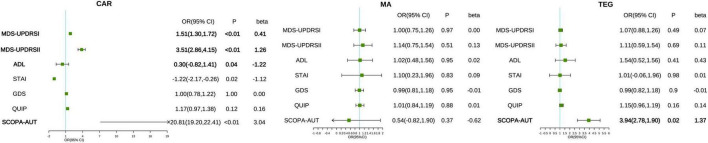
Associations between multimorbidity patterns with motor and neuropsychiatric scale scores. The correlation between the number of comorbidities and motor and neuropsychiatric symptoms in patients with prodromal Parkinson’s disease under different multimorbidity patterns. The pattern is adjusted based on age, gender, and educational level. The severity of motor and psychiatric symptoms is presented by MDS-UPDRS I, II, ADL, GDS, QUIP, and SCOPA-AUT. CAR, cardiometabolic multimorbidity pattern; MA, mental and arthritis multimorbidity pattern; TEG, Thyroid, eye, and gastrointestinal diseases pattern.

**FIGURE 3:**
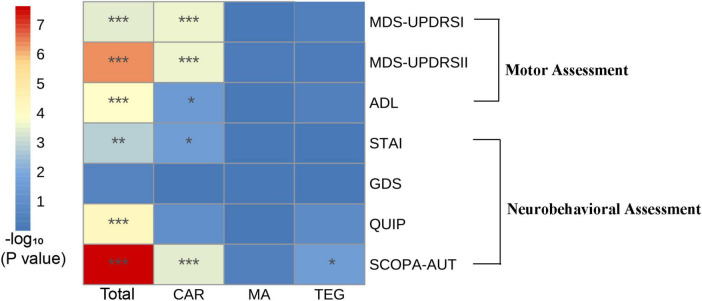
The relationship between the number of comorbidities and motor and neuropsychiatric symptoms in multimorbidity patterns. Comparison of the correlation between the three multimorbidity patterns and the overall population in various motor and neuropsychiatric scales. All of the outcomes were -log10 transformed. *P < 0.05; **P < 0.01; ***P < 0.001.

Three multimorbidity patterns has been identified at baseline: (1) Cardiometabolic multimorbidity pattern (CAR) [(113/416); (hypertension, hyperlipidemia, diabetes)]; (2) Mental and arthritis multimorbidity pattern (MA) [(198/416); (sleep disorder, depression anxiety, osteoporosis, inflammatory arthropathy)]; (3) Thyroid, eye, and gastrointestinal diseases pattern (TEG) [(105/416); (thyroid disease, eye disease, stomach disorder, cardiac valve diseases)] (Supplementary Table 3). Participants in CAR were more likely to be female, participants in MA were more likely to be male, and participants in TEG are more educated (Supplementary Table 2). In the three different multimorbidity patterns, different motor and neuropsychiatric scale scores were correlated with the number of comorbidities. The influence of CAR on motor and neuropsychiatry symptoms was more significant, and patients with increased number of comorbidities MDS-UPDRS I (*β* = 0.41, *P* < 0.001), MDS-UPDRS II (*β* = 1.26, *P* < 0.001), SCOPA-AUT (*β* = 3.04, *P* < 0.001) increased and ADL (*β* = −1.22, *P* = 0.04) decreased. However, STAI (*β* = −1.12, *P* = 0.02) was contrary to expectations, and the score was negatively correlated with the number of comorbidities. SCOPA-AUT (*β* = 1.37, *P* = 0.02) score also increased in patients with an increased number of comorbidities in TEG. No correlation was found between the scores of each scale and the number of comorbidities in MA (Table 4 and Figures 2, 3).

**TABLE 4:** Relationship between the number of comorbidities and symptoms in three multimorbidity patterns.

	Cardiometabolic multimorbidity pattern			Mental and arthritis multimorbidity pattern			Thyroid, eye, and gastrointestinal diseases pattern		
	***P***	***β***	**OR (95% CI)**	***P***	***β***	**OR (95% CI)**	***P***	***β***	**OR (95% CI)**
MDS-UPDRS I	< 0.001	0.41	1.51 (1.30, 1.72)	0.97	0.004	1.00 (0.75, 1.26)	0.49	0.07	1.07 (0.88, 1.26)
MDS-UPDRS II	< 0.001	1.26	3.51 (2.86, 4.15)	0.51	0.13	1.14 (0.75, 1.54)	0.69	0.11	1.11 (0.59, 1.54)
ADL	0.04	−1.22	0.30 (−0.82, 1.41)	0.95	0.02	1.02 (0.48, 1.56)	0.41	0.43	1.54 (0.52, 1.56)
STAI	0.02	−1.12	−1.22 (−2.17, −0.26)	0.83	0.09	1.10 (0.23, 1.96)	0.98	0.01	1.01 (−0.06, 1.96)
GDS	1.00	0.00	1.00 (0.78, 1.22)	0.95	−0.01	0.99 (0.81, 1.18)	0.90	−0.01	0.99 (0.82, 1.18)
QUIP	0.12	0.16	1.17 (0.97, 1.38)	0.88	0.01	1.01 (0.84, 1.19)	0.16	0.14	1.15 (0.96, 1.19)
SCOPA-AUT	< 0.001	3.04	20.81 (19.20, 22.41)	0.37	−0.62	0.54 (−0.82, 1.90)	0.02	1.37	3.94 (2.78, 1.90)

Relationship between the number of comorbidities and symptoms at baseline in patients with three multimorbidity patterns of non-Parkinson’s disease.

## 4 Discussion

In this study on Parkinson’s prodromal phase, we used the Parkinson’s Motor and Neuropsychiatric scale to study the cross-sectional and longitudinal correlation between Parkinson’s motor and psychiatric symptoms. We found that: (1) The increasing numbers of comorbidities were related to the faster progress of their motor and neuropsychiatric symptoms, which is specifically manifested in Parkinson’s symptoms of daily life, motor function, depression, and AS. (2) Compared with the other patterns, the CAR correlated more with motor and neuropsychiatric symptoms at baseline. The above association may be driven by strong correlations observed in severe multiple diseases (i.e., ≥ 4 chronic diseases). Our study proposed new opinions on the prevention and prediction of PD from the perspectives of the number of comorbidities and specific multimorbidity patterns.

### 4.1 Risk of multiple diseases and PD

In recent years, myocardial infarction, hypertension, diabetes, etc., were considered to increase the risk of PD (Qiu et al., 2011; Sun et al., 2012; Liang et al., 2015; Brauer et al., 2020). Gastrointestinal symptoms and dysregulation of gut microbes may precede the onset of Parkinson’s motor symptoms, and there had been many reports that the gut-brain axis played a role in PD (Metta et al., 2022). The main findings were consistent with previous evidence, and observational studies have shown that multiple diseases are associated with the occasion of PD or neurodegenerative diseases (Leibson et al., 2006; Santos Garcia et al., 2017). To comprehensively evaluate the multimorbidity status of participants, our study not only used quantitative approaches (reflected in the counting of comorbidities) but also captured clustering in patterns of multimorbidity. Compared with the non-comorbidity group (0–1 group), participants in the comorbidity group had a significantly increased danger of PD, indicating that the exist of two or more diseases may increase the risk of developing PD in patients. Multiple illnesses can reflect a worsening in physical condition. Comorbidities can reflect the deterioration of overall health status. There are studies have shown that the long-term coexistence of diseases is associated with neurodegenerative biomarkers and exacerbates brain pathology before the onset of dementia symptoms (Vassilaki et al., 2016).

The increase in comorbidity count was related to the increased in Parkinson’s risk. One possible explanation was that comorbidity caused small vascular disease and other cerebrovascular damage or promoted neurodegenerative processes through interaction with neurons or synapses at the cellular level (Roberts et al., 2014; Vassilaki et al., 2016). Second, chronic inflammation and oxidative stress in various chronic diseases may increase the danger risk of PD (for example myocardial infarction and diabetes) (Sun et al., 2012; Liang et al., 2015). Third, pathologically, PD was characterized as the loss of dopaminergic substantia nigra Striatum neurons with Lewy bodies (Qiu et al., 2011; Sveinbjornsdottir, 2016). Some chronic diseases (such as hypertension) lead to hypertensive vascular lesions in the basal ganglia (Greenberg et al., 2009), thalamus, and brain stem, which may affect the dopaminergic cells in the compact part and destroy the connection between substantia nigra neurons and the putamen part of the striatum (Qiu et al., 2011), increase the risk of PD. Finally, an increase in the number of comorbidities is related to an increased likelihood of multi-drug treatment and an increased burden of treatment, which may affect the brain and lead to nerve damage (Hu et al., 2022).

### 4.2 Motor symptoms

The clinical symptoms of PD were mainly defined by motor symptoms (Sveinbjornsdottir, 2016). Cardiovascular disease interacts with neurodegenerative disease, and comorbidity and alterable cardiovascular risk factors were related to the incidence of axial dyskinesia in PD (Kotagal et al., 2014). This study found that as the number of comorbidities increased, the motor symptoms disorders of PD patients became more pronounced. One explanation was that people with multiple diseases simultaneously were affected by different diseases, which increased the burden on their daily living abilities. Patients with multiple diseases were generally accompanied by multi-drug treatment, which increased the treatment burden and affected their motor symptoms. Another explanation was that the disease affected biomarkers associated with neurodegeneration in the brain, leading to the motor symptoms of PD. It had been previously studied that the long-term co-existence of the disease was related to neurodegenerative biomarkers in the brain (Kao et al., 2021; Weintraub et al., 2022). Among the influences on motor symptoms of PD, dopaminergic nigrostriatal neuronal degeneration in Louise’s body was considered to be the main neuropathologic correlation of motor disorders in PD. Before the main motor features of PD begin to appear, up to 80% of dopaminergic cells in the nigro-striatum system had been lost. PD was usually diagnosed by initial motor symptoms (Sveinbjornsdottir, 2016). Further research is needed in the future on whether multiple diseases can affect motor symptoms by affecting the dopaminergic nigrostriatal.

### 4.3 Neuropsychiatry symptom

The main clinical symptoms of Parkinson’s disease also include several other neurological disorders, and these symptoms may begin 10 years or earlier before diagnosis (Sveinbjornsdottir, 2016), increasing the possibility of depression and anxiety in patients with multiple diseases (Castro-de-Araujo et al., 2022; Felez-Nobrega et al., 2022). Compared to people without multiple diseases or chronic physical illness, people with multiple diseases had twice the risk of developing depression and three times the risk of people without chronic physical disease (Read et al., 2017). Anxiety and depression exist from the pre exercise phase of prodromal PD, anxiety is particularly dominant, and it is also common to overlap between anxiety and depression (Schapira et al., 2017). In PD patients, some observed signs and symptoms have a bidirectional risk association. Early onset of neuropsychiatric symptoms such as depression or anxiety in PD patients can also lead to accelerated progression of cognitive impairment (Forbes et al., 2021). The possibility of depression increases with the development of the disease (Gustafsson et al., 2015; Kazmi et al., 2021).

High doses and prolonged exposure to dopamine agonists were risk factors associated with ICDs. However, the timing of ICDs varies, as it depends on the use of dopaminergic medications, and the increased risk of ICDs was reported only in patients receiving dopaminergic therapy (Weintraub et al., 2022). Dopamine agonist therapy was associated with an increased danger of ICDs in PD patients (Weintraub et al., 2010). The prevalence of ICDs increases with the prolongation of the PD course (Markovic et al., 2020). The incidence of ICDs was not higher in newly diagnosed or untreated patients compared to the ordinary being (Weintraub et al., 2022). All patients in this study were non-Parkinson’s patients, so fewer patients received dopaminergic therapy. Another reason was that the inclusion of a small population in this study did not meet the conditions for discovering an association between the number of comorbidities and ICDs.

Our results demonstrated the role of comorbidity in AS in prodromal PD patients. AS may precede motor symptoms, and patients with higher scores on the SCOPA-AUT scale with more severe disease, longer duration of illness, and more significant motor impairment than patients with lower scores. Early PD patients support the idea that AS may precede motor symptoms, and these viewpoints will help identify PD patients in the premotor stage (Arnao et al., 2015).

### 4.4 Multimorbidity patterns

Our results suggested that different comorbidities correlate differently with motor and neuropsychiatric symptoms in prodromal PD patients. Assessing multiple diseases through patterns and recognizing their tendency to coexist with certain diseases. Among the 3 patterns studied in this study, CAR characterized by hypertension, hyperlipidemia, and diabetes was associated with a higher risk of PD motor symptoms and AS. Cardiovascular metabolic diseases and dementia had a cardio-brain connection; hypertension, obesity, and Hypercholesterolemia were associated with a high risk of dementia in later life, thus strengthening the possibility of etiological links (Potashkin et al., 2020). The interaction between neurodegeneration and cardiovascular dysfunction had been confirmed in dementia, and cardiovascular comorbidities were associated with axial movement disorders in PD (Kotagal et al., 2014). Hypertension can predict the progression rate of axial, non-axial, and total motor scores by affecting frontal leukoplakia lesions (Kotagal et al., 2014). Hyperglycemia can affect motor disorders in PD by affecting dopaminergic neurons in the substantia nigra and striatum (Sveinbjornsdottir, 2016; Potashkin et al., 2020).

There was a strong relationship between comorbidities and AS in patients with prodromal PD under CAR. Newly diagnosed PD patients had more gastrointestinal and cardiovascular problems, which also supports the view that PD may start from autonomic neuropathy (Verbaan et al., 2007). It was proved that patients with prodromal PD who suffer from multiple cardiometabolic diseases can indeed increase the risk of PD-related AS. There was also a strong correlation between cardiometabolic disease and depression (She et al., 2019). The diagnosis of diabetes patients increases the risk of depression and anxiety (Semenkovich et al., 2015; Buchberger et al., 2016). Although the TEG was only related to AS, it proved that it was not just the CAR, in patterns of other diseases (such as thyroid, eye, and gastrointestinal disorders), the psychotic symptoms of patients with prodromal PD were also associated with the disease. We admitted that anxiety and depression were present in participants with “MA,” one of the multimorbidity patterns, and their co-existence may affect the interaction of this pattern with neuropsychiatric symptoms.

In non-CAR patterns, such as MA, Many of the immune alterations associated with rheumatoid arthritis are now also implicated in depressive pathology and this association might explain in part the enhanced burden of depression in people with rheumatoid arthritis compared with the general population (Nerurkar et al., 2019). In TEG, Graves’ ophthalmopathy is the most common extrathyroid manifestation of Graves’s disease, and the thyrotropin receptor on orbital fibroblasts may be an important autoimmune target in the disease (Bahn, 2010). Hyperthyroidism is associated with cardiovascular disease, including mitral valve dysfunction (Ladenson, 1990). Aortic valve stenosis can cause gastrointestinal bleeding due to intestinal vascular dysplasia (Thompson et al., 2012). In the codisease model, diseases of different systems are grouped together due to pathological mechanisms or potential combinations based on chronic diseases. The combination of precursor characteristics and risk factors can be studied.

### 4.5 Limitations

Limitations of the study warrant consideration. First, some important clinical indicators were not included, such as cognitive function, cerebrospinal fluid, and neuroimaging. Second, the sample size of our research is relatively small, and five years was not enough to cover the whole course of PD. These associations need to be further investigated in the future with longitudinal studies with longer follow-up periods. Finally, we did not account for the relative severity of comorbidities. Therefore, future prospective studies are needed.

## 5 Conclusion

In summary, comorbidity affects motor and neuropsychiatry symptoms in prodromal Parkinson’s patients, which may precede PD symptoms. At the same time, PD motor and neuropsychiatry symptoms were more likely to occur in patients with CAR. Our findings suggested that rather than comorbidities and PD having a common mechanistic effect, comorbidities need to have a more devastating impact on PD survival to influence prognosis. The study comprehensively analyzed the impact of comorbidity burden on prodromal Parkinson’s motor and psychiatric symptoms, demonstrating the preventive and predictive value of comorbidity for PD. And it provided a more targeted disease for preventing PD – cardiovascular metabolic comorbidities.

## Data Availability

Publicly available datasets were analyzed in this study. This data can be found here: https://www.ppmi-info.org/data.

## Footnotes

1
http://www.r-project.org

## References

[B1] Aerqin Q. Chen X. T. Ou Y. N. Ma Y. H. Zhang Y. R. Hu H. Y. (2024). Associations between multimorbidity burden and Alzheimer’s pathology in older adults without dementia: The CABLE study. *Neurobiol. Aging* 134 1–8. 10.1016/j.neurobiolaging.2023.09.014 37950963

[B2] Arnao V. Cinturino A. Valentino F. Perini V. Mastrilli S. Bellavia G. (2015). In patient’s with Parkinson disease, autonomic symptoms are frequent and associated with other non-motor symptoms. *Clin. Auton. Res* .25 301–307. 26359270 10.1007/s10286-015-0306-x

[B3] Bahn R. S. (2010). Graves’ ophthalmopathy. *N. Engl. J. Med.* 362 726–738. 20181974 10.1056/NEJMra0905750 PMC3902010

[B4] Brauer R. Wei L. Ma T. Athauda D. Girges C. Vijiaratnam N. (2020). Diabetes medications and risk of Parkinson’s disease: A cohort study of patients with diabetes. *Brain* 143 3067–3076. 33011770 10.1093/brain/awaa262 PMC7794498

[B5] Buchberger B. Huppertz H. Krabbe L. Lux B. Mattivi J. T. Siafarikas A. (2016). Symptoms of depression and anxiety in youth with type 1 diabetes: A systematic review and meta-analysis. *Psychoneuroendocrinology* 70 70–84. 27179232 10.1016/j.psyneuen.2016.04.019

[B6] Castro-de-Araujo L. F. S. Rodrigues E. D. S. Machado D. B. Henriques C. M. P. Verotti M. P. Goncalves A. Q. (2022). Multimorbidity worsened anxiety and depression symptoms during the COVID-19 pandemic in Brazil. *J. Affect. Disord.* 314 86–93. 10.1016/j.jad.2022.07.005 35810830 PMC9259509

[B7] Cui J. Qin Y. Tian Y. Ge X. Han H. Yang Z. (2021). Activities of daily living as a longitudinal moderator of the effect of autonomic dysfunction on anxiety and depression of Parkinson’s patients. *Brain Behav.* 11:e2297. 10.1002/brb3.2297 34333879 PMC8413789

[B8] Fan J. Sun Z. Yu C. Guo Y. Pei P. Yang L. (2022). Multimorbidity patterns and association with mortality in 0.5 million Chinese adults. *Chin. Med. J. (Engl)* 135 648–657. 35191418 10.1097/CM9.0000000000001985 PMC9276333

[B9] Felez-Nobrega M. Haro J. M. Koyanagi A. (2022). Multimorbidity, depression with anxiety symptoms, and decrements in health in 47 low- and middle-income countries. *J. Affect. Disord.* 317 176–184. 10.1016/j.jad.2022.08.110 36055525

[B10] Forbes E. Tropea T. F. Mantri S. Xie S. X. Morley J. F. (2021). Modifiable comorbidities associated with cognitive decline in Parkinson’s disease. *Mov. Disord. Clin. Pract.* 8 254–263. 33553496 10.1002/mdc3.13143 PMC7853194

[B11] GBD 2015 Disease and Injury Incidence and Prevalence Collaborators (2016). Global, regional, and national incidence, prevalence, and years lived with disability for 310 diseases and injuries, 1990-2015: A systematic analysis for the Global Burden of Disease Study 2015. *Lancet* 388 1545–1602. 27733282 10.1016/S0140-6736(16)31678-6 PMC5055577

[B12] Goetz C. G. Fahn S. Martinez-Martin P. Poewe W. Sampaio C. Stebbins G. T. (2007). Movement Disorder Society-sponsored revision of the Unified Parkinson’s Disease Rating Scale (MDS-UPDRS): Process, format, and clinimetric testing plan. *Mov. Disord.* 22 41–47. 10.1002/mds.21198 17115387

[B13] Goetz C. G. Tilley B. C. Shaftman S. R. Stebbins G. T. Fahn S. Martinez-Martin P. (2008). Movement Disorder Society-sponsored revision of the Unified Parkinson’s Disease Rating Scale (MDS-UPDRS): Scale presentation and clinimetric testing results. *Mov. Disord.* 23 2129–2170. 19025984 10.1002/mds.22340

[B14] Grande G. Marengoni A. Vetrano D. L. Roso-Llorach A. Rizzuto D. Zucchelli A. (2021). Multimorbidity burden and dementia risk in older adults: The role of inflammation and genetics. *Alzheimers Dement.* 17 768–776. 10.1002/alz.12237 33403740 PMC8247430

[B15] Greenberg S. M. Vernooij M. W. Cordonnier C. Viswanathan A. Al-Shahi Salman R. Warach S. (2009). Cerebral microbleeds: A guide to detection and interpretation. *Lancet Neurol.* 8 165–174. 19161908 10.1016/S1474-4422(09)70013-4 PMC3414436

[B16] Guisado-Clavero M. Roso-Llorach A. Lopez-Jimenez T. Pons-Vigues M. Foguet-Boreu Q. Munoz M. A. (2018). Multimorbidity patterns in the elderly: A prospective cohort study with cluster analysis. *BMC Geriatr.* 18:16. 10.1186/s12877-018-0705-7 29338690 PMC5771078

[B17] Gustafsson H. Nordstrom A. Nordstrom P. (2015). Depression and subsequent risk of Parkinson disease: A nationwide cohort study. *Neurology* 84 2422–2429. 25995056 10.1212/WNL.0000000000001684 PMC4478031

[B18] Hu H. Y. Zhang Y. R. Aerqin Q. Ou Y. N. Wang Z. T. Cheng W. (2022). Association between multimorbidity status and incident dementia: A prospective cohort study of 245,483 participants. *Transl. Psychiatry* 12:505. 10.1038/s41398-022-02268-3 PMC9729184 36476644

[B19] Kao S. L. Wang J. H. Chen S. C. Li Y. Y. Yang Y. L. Lo R. Y. (2021). Impact of comorbidity burden on cognitive decline: A Prospective Cohort Study of older adults with dementia. *Dement. Geriatr. Cogn. Disord.* 50 43–50. 33789290 10.1159/000514651

[B20] Kazmi H. Walker Z. Booij J. Khan F. Shah S. Sudre C. H. (2021). Late onset depression: Dopaminergic deficit and clinical features of prodromal Parkinson’s disease: A cross-sectional study. *J. Neurol. Neurosurg. Psychiatry* 92 158–164. 10.1136/jnnp-2020-324266 33268471 PMC7841491

[B21] Kotagal V. Albin R. L. Muller M. L. Koeppe R. A. Frey K. A. Bohnen N. I. (2014). Modifiable cardiovascular risk factors and axial motor impairments in Parkinson disease. *Neurology* 82 1514–1520. 24682965 10.1212/WNL.0000000000000356 PMC4011463

[B22] Ladenson P. W. (1990). Recognition and management of cardiovascular disease related to thyroid dysfunction. *Am. J. Med.* 88 638–641. 2189308 10.1016/0002-9343(90)90532-i

[B23] Leelakanok N. D’Cunha R. R. (2019). Association between polypharmacy and dementia - A systematic review and metaanalysis. *Aging Ment. Health* 23 932–941. 29746153 10.1080/13607863.2018.1468411

[B24] Leibson C. L. Maraganore D. M. Bower J. H. Ransom J. E. O’Brien P. C. Rocca W. A. (2006). Comorbid conditions associated with Parkinson’s disease: A population-based study. *Mov. Disord.* 21 446–455. 16161155 10.1002/mds.20685

[B25] Liang H. W. Huang Y. P. Pan S. L. (2015). Parkinson disease and risk of acute myocardial infarction: A population-based, propensity score-matched, longitudinal follow-up study. *Am. Heart J.* 169 508–514. 10.1016/j.ahj.2014.11.018 25819857

[B26] Markovic V. Stankovic I. Petrovic I. Stojkovic T. Dragasevic-Miskovic N. Radovanovic S. (2020). Dynamics of impulsive-compulsive behaviors in early Parkinson’s disease: A prospective study. *J. Neurol.* 267 1127–1136. 10.1007/s00415-019-09692-4 31902006

[B27] Metta V. Leta V. Mrudula K. R. Prashanth L. K. Goyal V. Borgohain R. (2022). Gastrointestinal dysfunction in Parkinson’s disease: Molecular pathology and implications of gut microbiome, probiotics, and fecal microbiota transplantation. *J. Neurol.* 269 1154–1163. 33881598 10.1007/s00415-021-10567-w

[B28] Nerurkar L. Siebert S. McInnes I. B. Cavanagh J. (2019). Rheumatoid arthritis and depression: An inflammatory perspective. *Lancet Psychiatry* 6 164–173. 30366684 10.1016/S2215-0366(18)30255-4

[B29] Potashkin J. Huang X. Becker C. Chen H. Foltynie T. Marras C. (2020). Understanding the links between cardiovascular disease and Parkinson’s disease. *Mov. Disord.* 35 55–74. 31483535 10.1002/mds.27836 PMC6981000

[B30] Qiu C. Hu G. Kivipelto M. Laatikainen T. Antikainen R. Fratiglioni L. (2011). Association of blood pressure and hypertension with the risk of Parkinson disease: The National FINRISK Study. *Hypertension* 57 1094–1100. 10.1161/HYPERTENSIONAHA.111.171249 21536985

[B31] Read J. R. Sharpe L. Modini M. Dear B. F. (2017). Multimorbidity and depression: A systematic review and meta-analysis. *J. Affect. Disord.* 221 36–46. 28628766 10.1016/j.jad.2017.06.009

[B32] Regnault A. Boroojerdi B. Meunier J. Bani M. Morel T. Cano S. (2019). Does the MDS-UPDRS provide the precision to assess progression in early Parkinson’s disease? Learnings from the Parkinson’s progression marker initiative cohort. *J. Neurol.* 266 1927–1936. 10.1007/s00415-019-09348-3 31073716 PMC6647182

[B33] Roberts R. O. Knopman D. S. Przybelski S. A. Mielke M. M. Kantarci K. Preboske G. M. (2014). Association of type 2 diabetes with brain atrophy and cognitive impairment. *Neurology* 82 1132–1141. 24647028 10.1212/WNL.0000000000000269 PMC3966799

[B34] Santos Garcia D. Suarez Castro E. Exposito I. de Deus T. Tunas C. Aneiros A. (2017). Comorbid conditions associated with Parkinson’s disease: A longitudinal and comparative study with Alzheimer disease and control subjects. *J. Neurol. Sci.* 373 210–215. 10.1016/j.jns.2016.12.046 28131190

[B35] Schapira A. H. V. Chaudhuri K. R. Jenner P. (2017). Non-motor features of Parkinson disease. *Nat. Rev. Neurosci.* 18 435–450. 28592904 10.1038/nrn.2017.62

[B36] Semenkovich K. Brown M. E. Svrakic D. M. Lustman P. J. (2015). Depression in type 2 diabetes mellitus: Prevalence, impact, and treatment. *Drugs* 75 577–587. 25851098 10.1007/s40265-015-0347-4

[B37] She R. Yan Z. Jiang H. Vetrano D. L. Lau J. T. F. Qiu C. (2019). Multimorbidity and health-related quality of life in old age: Role of functional dependence and depressive symptoms. *J. Am. Med. Dir. Assoc.* 20 1143–1149. 10.1016/j.jamda.2019.02.024 30979676

[B38] Sun Y. Chang Y. H. Chen H. F. Su Y. H. Su H. F. Li C. Y. (2012). Risk of Parkinson disease onset in patients with diabetes: A 9-year population-based cohort study with age and sex stratifications. *Diabetes Care* 35 1047–1049. 10.2337/dc11-1511 22432112 PMC3329814

[B39] Sveinbjornsdottir S. (2016). The clinical symptoms of Parkinson’s disease. *J. Neurochem.* 139 Suppl. 1 318–324. 27401947 10.1111/jnc.13691

[B40] Thompson J. L. III Schaff H. V. Dearani J. A. Park S. J. Sundt T. M. III Suri R. M. (2012). Risk of recurrent gastrointestinal bleeding after aortic valve replacement in patients with Heyde syndrome. *J. Thorac. Cardiovasc. Surg.* 144 112–116. 21864855 10.1016/j.jtcvs.2011.05.034

[B41] Vassilaki M. Aakre J. A. Mielke M. M. Geda Y. E. Kremers W. K. Alhurani R. E. (2016). Multimorbidity and neuroimaging biomarkers among cognitively normal persons. *Neurology* 86 2077–2084. 27164657 10.1212/WNL.0000000000002624 PMC4891208

[B42] Verbaan D. Marinus J. Visser M. van Rooden S. M. Stiggelbout A. M. van Hilten J. J. (2007). Patient-reported autonomic symptoms in Parkinson disease. *Neurology* 69 333–341. 17646625 10.1212/01.wnl.0000266593.50534.e8

[B43] Weintraub D. Aarsland D. Chaudhuri K. R. Dobkin R. D. Leentjens A. F. Rodriguez-Violante M. (2022). The neuropsychiatry of Parkinson’s disease: Advances and challenges. *Lancet Neurol.* 21 89–102. 34942142 10.1016/S1474-4422(21)00330-6 PMC8800169

[B44] Weintraub D. Koester J. Potenza M. N. Siderowf A. D. Stacy M. Voon V. (2010). Impulse control disorders in Parkinson disease: A cross-sectional study of 3090 patients. *Arch. Neurol.* 67 589–595. 10.1001/archneurol.2010.65 20457959

