# Supplementary material for: Associations of motor and neuropsychiatric symptoms with comorbidities in prodromal Parkinson’s disease
Source: Front Aging Neurosci. 2024 Nov 25;16:1452766. doi: 10.3389/fnagi.2024.1452766 (PMC11625736; doi:10.3389/fnagi.2024.1452766)
Supplement: Supplementary file 1 [file Data_Sheet_1.docx]

**Supplementary Table 1：MDS-UPDRS Ⅳ crowd characteristics.**

| **Variables（Mean ± SD）** | **Total**  **(N = 148)** | **0~1**  **(N = 61)** | **2~3**  **(N = 58)** | **≥4**  **(N = 29)** | **p** |
| --- | --- | --- | --- | --- | --- |
| **AGE** | 62.21 ± 9.22 | 58.82±8.64 | 63.24±8.95 | 67.27±8.37 | <0.001 |
| **SEX, n (%)** |  |  |  |  | 0.688 |
| **male** | 65(43.9) | 28(45.9) | 23(39.7) | 14(48.3) |  |
| **female** | 83(56.1) | 33(54.1) | 35(60.3) | 15(51.7) |  |
| **Education, years** | 16.15±3.86 | 15.57±4.19 | 16.40±3.50 | 16.86±3.76 | 0.237 |
| **MDS-UPDRS Ⅳ** | 0.12±0.65 | 0.15±0.75 | 0.07±0.53 | 0.17±0.66 | 0.481 |

Data are summarized as mean ± standard deviation (SD) for continuous data and count (%) for categorical data. Kruskal-Wallis test was used for continuous variables and Pearson Chi-square test was used for categorical variables.

Abbreviations: N, number; MDS-UPDRS Ⅳ, Movement Disorders Society Unified Parkinson Disease Rating Scale part Ⅳ

**Supplementary Figure 1:** **Flowchart of participant selection and the research process.**


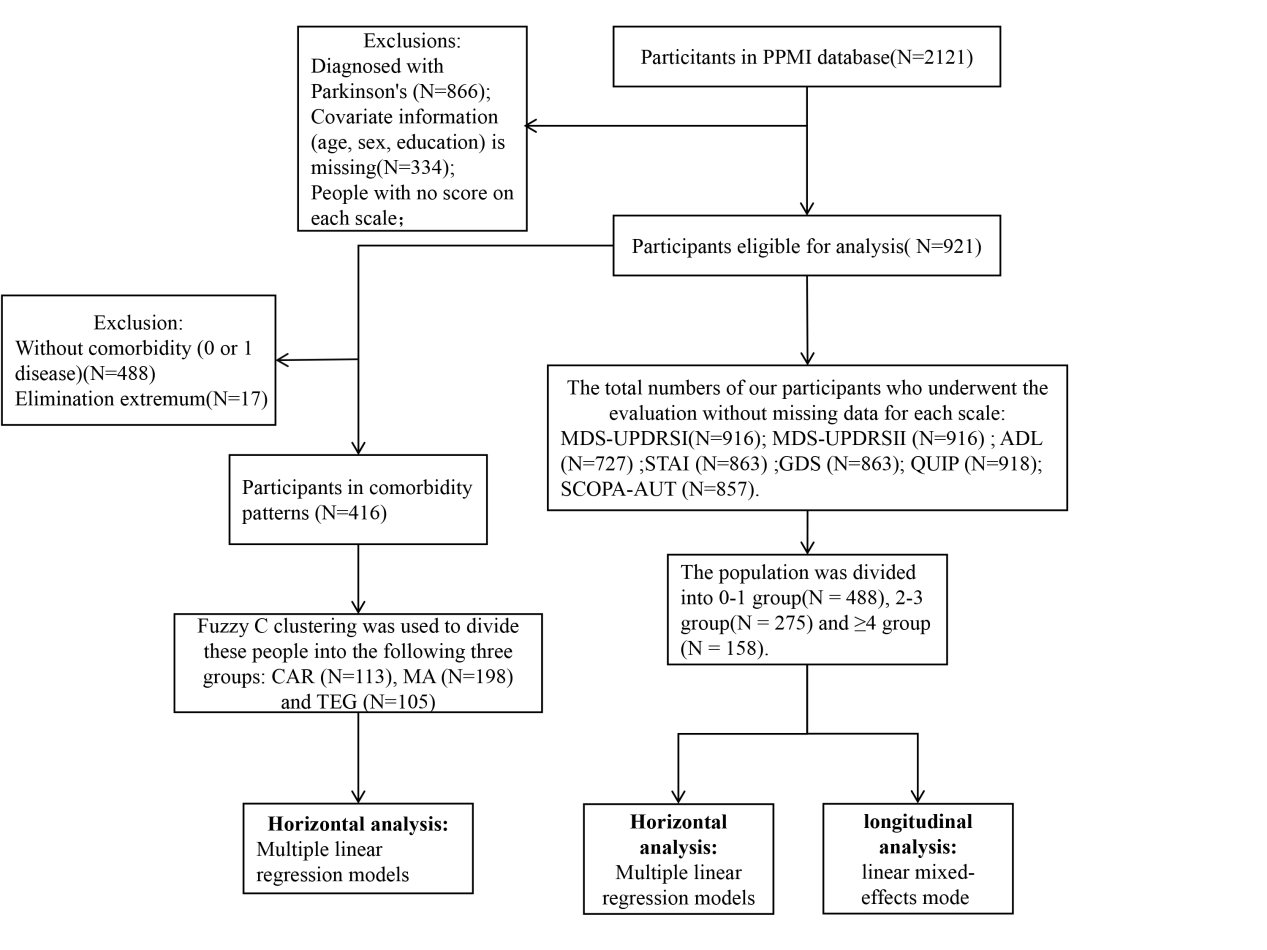


**Supplementary Table 2. Baseline characteristics of participants by number of Multimorbidity patterns**

|  | **Total**  **(N=416)** | **Cardiometabolic multimorbidity pattern(CAR)**  **(N=113)** | **Mental and arthritis multimorbidity pattern(MA)**  **(N=198)** | **Thyroid, eye, and gastrointestinal diseases pattern(TEG)**  **(N=105)** | **P** |
| --- | --- | --- | --- | --- | --- |
| **AGE** | 64.28 ± 8.07 | 65.63±7.28^b^ | 65.52±8.27^ac^ | 66.14±7.87^b^ | < 0.001 |
| **SEX, N (%)** |  |  |  |  | < 0.001 |
| **male** | 217 (52.16%) | 37(32.74%) | 134(67.68%) | 46(43.81%) |  |
| **female** | 199 (47.84%) | 76(67.26%) | 64(32.32%) | 59(56.19%) |  |
| **Education, years** | 16.75 ± 3.52 | 15.98±3.95^c^ | 16.88±3.54 | 17.30±2.80^a^ | 0.021 |

Data are summarized as mean ± standard deviation (SD) for continuous data and count (%) for categorical data. ^a^ Significantly different from CAR group; ^b^ Significantly different from MA group; ^c^ Significantly different from TEG group.

**Supplementary Table 3：** **The selection of diseases in various multimorbidity patterns.**

| **Multimorbidity patterns**  **(N=416)** | **Disease** | **O/E ratio** | **Exclusivity**  **(%)** | **Proportion within**  **the pattern (%)** |
| --- | --- | --- | --- | --- |
| **CAR**  **(N=113)** | Hypertension | 1.77 | 48.02% | 85.84% |
|  | Hyperlipidemia | 1.47 | 39.90% | 68.14% |
|  | Diabetes | 1.92 | 52.11% | 32.74% |
| **MA**  **(N=198)** | Sleep disorder | 1.50 | 71.31% | 43.94% |
|  | Depression anxiety | 1.70 | 81.05% | 77.78% |
|  | Osteoporosis | 1.48 | 70.37% | 19.19% |
|  | Inflammatory arthropathy | 1.01 | 48.10% | 19.19% |
| **TEG**  **(N=105)** | Cardiac valve diseases | 1.91 | 48.10% | 36.19% |
|  | Thyroid diseases | 1.95 | 49.19% | 86.67% |
|  | Eye disease | 1.18 | 29.82% | 16.19% |
|  | Stomach disorder | 1.10 | 27.84% | 25.71% |

O/E ratio is calculated by dividing the prevalence of a particular disease in a group by its prevalence in the general population; Exclusivity defined as the proportion of participants with the disease included in the cluster to the total number of participants with the disease.

Abbreviations: CAR, cardiometabolic multimorbidity pattern; MA, Mental and arthritis multimorbidity pattern; TEG, Thyroid, eye, and gastrointestinal diseases pattern; O/E ratio, observed/expected ratio.
